# Photo-Rheological Fluid-Based Colorimetric Ultraviolet Light Intensity Sensor

**DOI:** 10.3390/s19051128

**Published:** 2019-03-05

**Authors:** Kyung-Pyo Min, Gi-Woo Kim

**Affiliations:** Department of Mechanical Engineering, Inha University, Incheon 22212, Korea; min20you@naver.com

**Keywords:** photo-rheological (PR) fluids, UV light-responsive, UV intensity sensor, digital image processing

## Abstract

This study presents an introduction to a new type of ultraviolet (UV) light intensity sensor using photo-rheological (PR) fluids whose properties, such as color, can be changed by UV light. When the PR fluids were irradiated by UV light, colorimetric transitions were observed. Effectively, this means that their color changed gradually from yellow to red. The degree of the color change depended on the UV light intensity and was characterized by the hue value of the images acquired with a compact image sensor. We demonstrated that UV light-responsive capabilities can be readily imparted to PR fluids, and that the colorimetric responses to different UV light intensities can be used to measure the UV light intensities.

## 1. Introduction

Photo-rheological (PR) fluids are fluids that exhibit changes in their viscosity upon ultraviolet (UV) light irradiation. These fluids have attracted increased attention since Lee et al. reported that they can be easily synthesized in the laboratory using commercially available chemicals and ingredients, such as spiropyran, instead of using conventional ingredients, such as trans-ortho-methoxycinnamic acid, alpha-cyclodextrin, and hydroxyethyl cellulose [1,2,3,4]. PR fluids are characterized by changes in their properties such as color and viscosity when irradiated by the UV light [5]. A study by Cho et al. demonstrated the possibility of implementing damper and flow valves based on the principle of viscosity changes of PR fluids [5,6]. Meanwhile, Jin et al. experimented with PR fluids to verify the dual-response characteristic evoked by UV light and temperature [7].

Typically, UV sensors are implemented using semiconductors made of ZnO and ZnS, which have wide bandgaps and increased sensitivities to UV light [8]. A study by Zhai et al. compared the properties of oxide metals used for UV sensor fabrication [9]. Accordingly, many engineering studies improved the properties of UV sensors, including their sensitivity, stability, and efficiency, by altering or synthesizing the structure of the material [10,11,12,13]. However, ZnO and ZnS can only detect UV wavelengths in the range of 320 nm to 400 nm because of the inherent limitations of UV-sensitive materials [8]. Thus, nitride semiconductors, such as GaN, InN, and AlN, have been developed to detect shorter UV light wavelengths [14,15,16]. A study by Zheng et al. presented a UV sensor that could detect short UV wavelengths below 200 nm using AlN micro/nanowires and compared the parameters of extensively used semiconductors in UV sensors [15]. In addition, many engineering studies have been conducted on UV sensors with various materials, such as titanium dioxide or diamond, to improve the performance of UV sensors [17,18,19,20,21]. In particular, niobium pentoxide has a much higher sensitivity and external quantum efficiency than some other employed materials, and it has been extensively studied as a UV sensor [17]. The aforementioned UV sensors can measure UV light exactly, they have fast responses, and the range of wavelengths that can be detected is broad. However, most of the aforementioned sensors require complicated electronic circuits and peripherals because they use semiconductors and respond to UV light electronically. However, the proposed sensor can measure UV intensity colorimetrically, which implies that it does not require any additional electronic circuits. In the case of pH and temperature, Lee et al. demonstrated a sensor in a colorimetric system that used the polydiacetylenes (PDA) and confirmed a colorimetric response to UV light [22]. In addition, colorimetric sensors are being developed using various materials and methods besides PDA. Recently, a colorimetric UV sensor was synthesized using gallium oxide that responded even to short UV wavelengths at ~254 nm [23]. Furthermore, colorimetric gas sensors that were based on continuous monitoring have been used to detect ozone gas [24]. However, the colorimetric responses of these sensors were only qualitative (visual) and not quantitative (numerical). To express the color values numerically, the colorimetric response should be represented as a value in the color space using digital image processing. There are various types of color spaces, such as the RGB, HSV (hue, saturation, value), and CIELAB (also known as CIE L*a*b). The color space HSV is associated with a higher efficiency for color texture analyses, as compared to the other color spaces in normal states (i.e., no noise) and in noisy conditions [25,26]. A study by Cantrell et al. researched the optical sensor using the hue parameter of the HSV color space because this parameter was stable and quantitative [27]. However, to the best of our knowledge, no prior study has attempted to utilize the color change of PR fluids with the use of digital image processing.

Therefore, the primary objective of this study is to demonstrate the possibility of implementing a colorimetric UV intensity sensor by demonstrating the colorimetric responses of PR fluids (i.e., their color changes) and by characterizing them using a digital image processing technique. In Section 2, the fundamental properties and preparation of PR fluids are briefly reviewed. A detailed description of the mechanism of the proposed sensor and the digital image processing algorithm are introduced in Section 3. The UV light intensity measurements and preliminary results are presented in Section 4. The linear relationship between the UV intensity and the color changes of PR fluid have been demonstrated based on laboratory test-bed experiments.

## 2. Overview of PR Fluids

**Figure 1:**
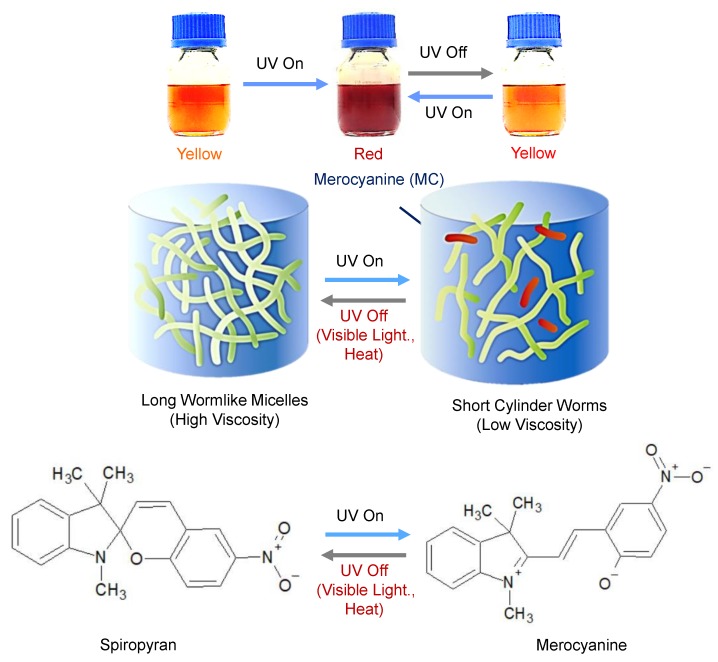
Photo-rheological (PR) phenomenon of wormlike reverse micelle-based PR fluid and the chemical structures of spiropyran (SP) and merocyanine.

The PR phenomenon originated from the interaction between the phospholipid lecithin and sodium deoxycholate (SDC) in an organic solvent, such as cyclohexane [1]. Before UV irradiation, spiropyran (SP) remained in the hydrophobic state in the presence of SDC, and the head group of lecithin interacted with SDC. Typically, the hydrophobic tails of lecithin acted as networks, possessing wormlike-shaped micelles, as shown in Figure 1. Upon UV irradiation, the SP was converted to a hydrophilic state in the presence of merocyanine (MC), and shift to the head-group area of lecithin. This SP-doped reverse micelles-based chemical shift led to the color change of the PR fluid from yellow to red. The reduction in the head-group size resulted in a cone-shaped molecular geometry. In turn, the self-assembly was transformed into shorter cylindrical worms that reduced the viscosity and elicited the referred color change. The preparation process of the PR fluids can be summarized as follows: The lecithin (7.8 g), and SDC (1.65 g) were first mixed in cyclohexane (100 mL). The solution then became transparent and homogeneous by magnetic stirring and heating at temperature of 65 °C until it was fully melted. The sample was then cooled to an ambient temperature (40 °C), and the SP (0.48 g) was added. This mixture was stirred until the SP was fully dissolved in the mixed solution. More detailed information on the preparation of the PR fluid sample based on SP-doped reverse micelles from commercially available products is described in the literature [1]. The three primary ingredients of the PR fluid samples synthesized for the colorimetric UV intensity sensor are listed in Table 1.

## 3. Working Principle of Colorimetric UV Intensity Sensor

### 3.1. Design of Colorimetric UV Intensity Sensor

**Figure 2:**
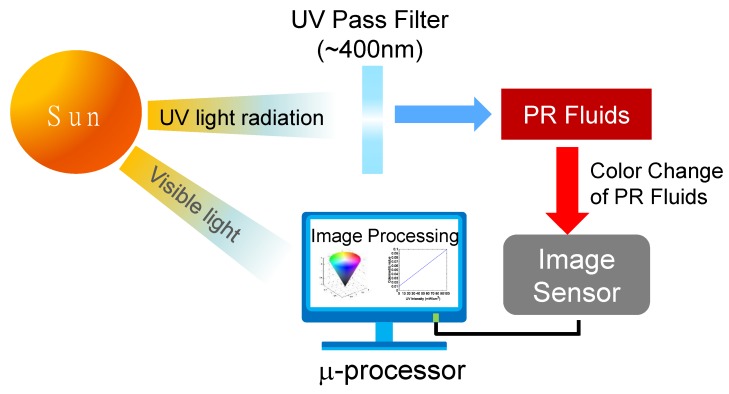
Measurement principle of colorimetric UV intensity sensor.

The measurement principle of the colorimetric UV intensity sensor is described in Figure 2. When UV light is irradiated on a PR fluid sample, the color of the PR fluid is changed from yellow to red. Unlike the conventional color sensors using surface characteristics, such as reflectance and transmittance (analog type), the color change produced by UV light is directly captured by an image sensor such as a CMOS-type image sensor. Subsequently, the colorimetric values, such as hue, brightness, and saturation, are obtained using a digital image processing technique. The colorimetric values are different depending on the UV intensity. Accordingly, the UV intensity can be measured based on sensitivity curves of the UV intensities, and the values obtained from the digital image processing. Given that the measurement of the UV light intensity only requires an image sensor, this leads to a simple structure, and increased cost-effectiveness. Accordingly, this sensor can be advantageous over conventional UV light intensity sensors.

### 3.2. Digital Image Processing Algorithm

**Figure 3:**
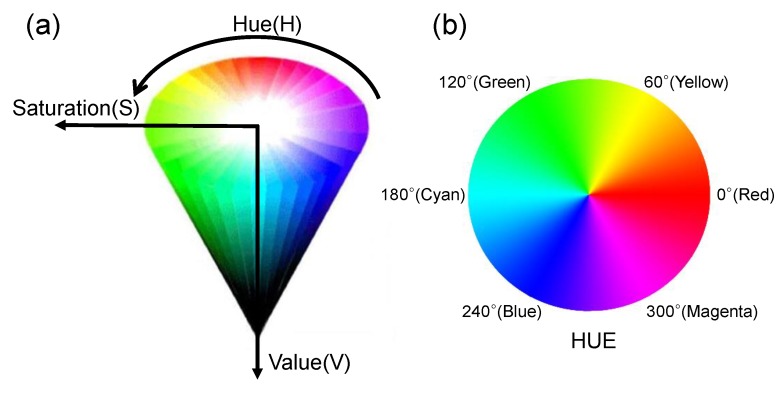
Representation of the HSV (hue, saturation, value), color space (**a**) schematic and (**b**) color circle in the hue domain.

Typically, when a color is represented numerically, it is expressed using the coordinates of the color space. It is difficult to represent the type of color with single variables in the RGB space because the RGB color space represents all the colors based on the combination of red (R), green (G), and blue (B). Unlike the conventional color sensor that senses the RGB and converts to digital values, the HSV color space is used to sense the color change because any color is represented using three variables in the HSV space: hue (H), saturation (S), and value (V), which are more suitable for sensing the color (single variable). As shown in Figure 3, the HSV color space represents the type of color (e.g., yellow) by using only the hue parameter. Accordingly, the hue parameter in the HSV color space is more suitable for the numerical representation of the measured values in colorimetric sensor systems [27]. The hue parameter can be easily obtained by converting from the RGB color space into the HSV color space using Equations (1) and (2) [28]. The hue parameter is then typically expressed as an angle between 0° and 360°, as shown in Figure 3b.

(1) $$\begin{array}{c} m_{\max}=\max\left(R,G,B\right)\\ m_{\min}=\min\left(R,G,B\right)\\ \Delta =m_{\max}-m_{\min} \end{array}$$

(2) $$\begin{array}{l} H=\{\begin{array}{cc} \frac{G-B}{\Delta }\times 60 & \mathrm{if}\;m_{\max}=R\;\\ \left(2+\frac{B-R}{\Delta }\right)\times 60 & \mathrm{if}\;m_{\max}=G\\ \left(4+\frac{R-G}{\Delta }\right)\times 60 & \mathrm{if}\;m_{\max}=B \end{array}\\ H=\begin{array}{cc} H+360 & \mathrm{if}\;\;H<0 \end{array} \end{array}$$

The color circle in the hue domain (i.e., 0°~360°), is typically normalized to the range of 0–1. As a result, when the color of PR fluids is changed from yellow to red after UV light irradiation, the hue values of the PR fluids are decreased from about 0.15° to 0° because the hue value of the yellow color is located at 60° and the pure red color is located at 0°. Accordingly, the UV light intensity can be measured by extracting the hue values from the digital images. The region of interest (ROI) is cropped from the original images. The RGB value for each of the pixels of the cropped image is then extracted and converted to the hue value using Equations (1) and (2). Finally, the hue values of all pixels are averaged to generate the one hue value, and take the reciprocal of the one hue value.

## 4. Experimental Validation

### 4.1. Experimental Setup

**Figure 4:**
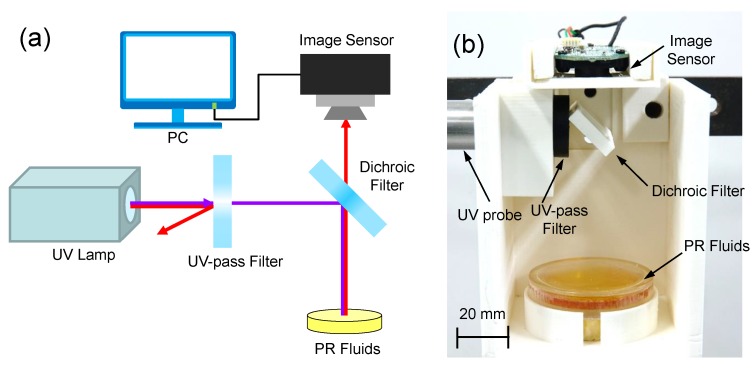
Experimental setup for measuring the UV light intensity: (**a**) schematic; (**b**) photograph.

The feasibility of the proposed UV light intensity sensor was validated based on experiments using the prototype sensor, as shown in Figure 4 [29]. Although the focal length of the UV lamp was 15 mm, the UV lamp, located at a constant distance of 60 mm, was used to change the color of the PR fluid. The dichroic optical filter (Edmond Optics 86–316) was used to selectively transmit visible light and reflect other wavelengths. The UV light intensity was increased from 0 to $2000\;\mathrm{mW}/cm^{2}$, and the color image of the PR fluids was captured by a CMOS-type image sensor, which has the optical resolution of 1280 × 960 pixels, fixed-focus type, and the focal length of 4.0 mm. When the UV light was irradiated on the PR fluids, the exposure time was set to 3 s. The capsule of the PR fluids was fabricated with the use of a transparent polycarbonate. The upper surface was also covered by a transparent polycarbonate after it was filled with the PR fluid (thickness of 2 mm), and an epoxy adhesive was used to bond them tightly.

**Figure 5:**
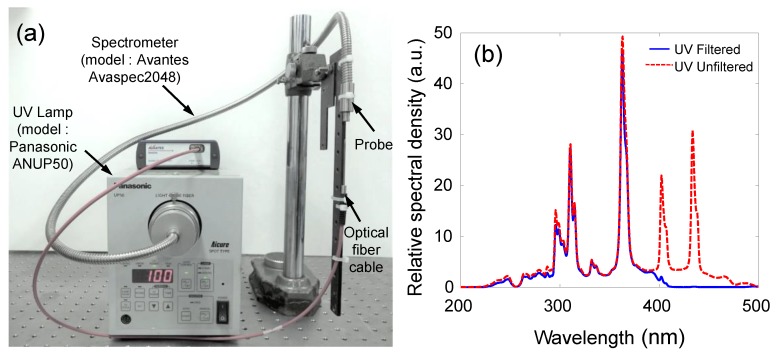
UV light source for experiment: (**a**) UV lamp and spectrometer and (**b**) spectral responses.

In this study, a commercial UV lamp (model: Panasonic ANUP50) was used as a UV light radiation source. The intensity and wavelength of the UV light from the UV lamp were measured immediately to assess the irradiation from the UV lamp using a spectrometer (Avaspec2048, Avantes, Apeldoorn, Netherlands). The wavelength spectra yielded an intense peak at a UV light wavelength value of 365 nm, as shown in Figure 5.

**Figure 6:**
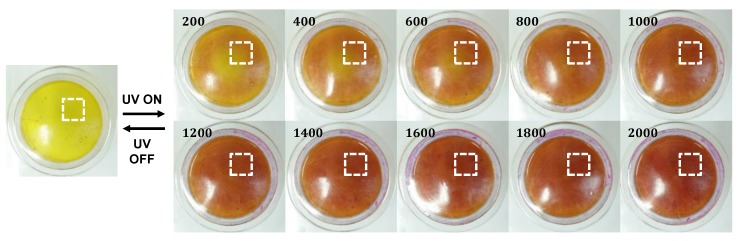
Colorimetric responses of PR fluids at different UV light intensities (unit: $\mathrm{mW}/cm^{2}$) at ambient temperature.

**Figure 7:**
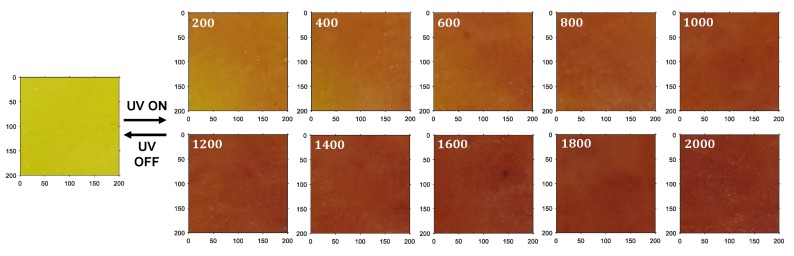
Cropped images from original colorimetric responses for digital image processing.

When the intensity of the UV light was increased up to 2000 $\mathrm{mW}/cm^{2}$, the colorimetric transitions were observed. In other words, the colors of the PR fluids gradually changed from yellow to red, as shown in Figure 6. The PR fluid inside the capsule exhibited a yellow color when there was no UV light irradiation, and changed to a pure red color at 2000 $\mathrm{mW}/cm^{2}$. Collectively, these responses are referred to as colorimetric responses. The original images were cropped at the ROI (dashed rectangular lines in Figure 6) with a size of 200×200 pixels before digital image processing, as shown in Figure 7. This experiment was performed in real time using a PC (TUF FX504, ASUS, Taipei, Taiwan) (Figure 4a) and with the MATLAB/Image Processing Toolbox provided by MathWorks^TM^, Natick, MA, USA) [30].

### 4.2. Results and Discussion

**Figure 8:**
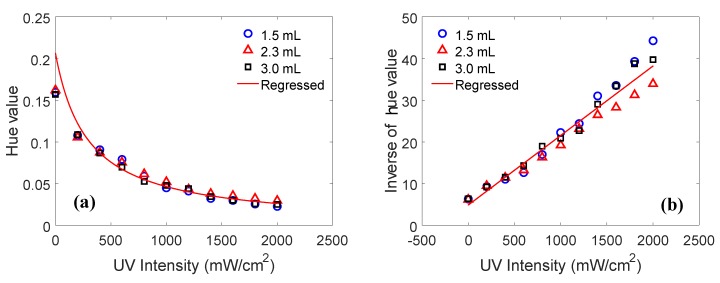
Measured sensitivity curves: (**a**) hue value and (**b**) inverse of the hue value ($R^{2}=0.991)$.

In this study, three different volumes of capsule (1.5 mL, 2.3 mL, and 3.0 mL) were used to investigate the effect of capsule size on the hue value. The hue values were then extracted from the cropped images (Figure 7) with the proposed digital image processing algorithm. Similar to semiconductor materials that exhibit resistance changes upon changes in a physical quantity (e.g., temperature in a thermistor), the hue value was inversely proportional to the UV light intensities (Figure 8a). As a result, the inverse of the hue values versus the UV intensity exhibited a linear response because the hue value was inversely proportional to the UV intensity. The three hue values were averaged at different UV intensities and regressed (curve fitted) by the following formulae (red solid line in Figure 8b):

(3) $$y_{1}=\frac{1}{0.0167x+4.844}$$

(4) $$y_{2}=0.0167x+4.844$$

where $y_{1}$ denotes the hue value of PR fluids, $y_{2}$ denotes the inverse of the hue value, and *x* denotes UV light intensity ($\mathrm{mW}/cm^{2}$). 

Although the hue values exhibited some differences at a higher UV intensity (beyond 1500 $\mathrm{mW}/cm^{2})$, these linear responses provided the good initial assessment information for the development of a new UV light intensity sensor.

**Figure 9:**
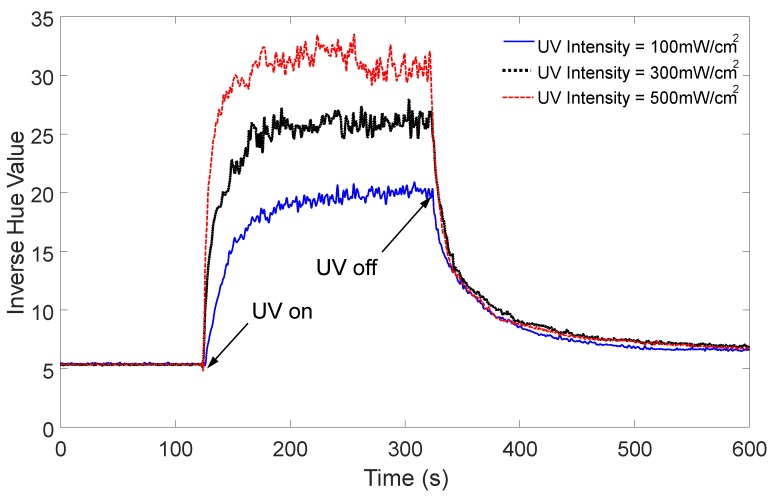
Transient response of inverse hue value at different UV light intensities.

The response time required to reach the steady state was then investigated from the transient response of the inverse hue value at different UV light intensities, as shown in Figure 9. While the rising time ranged from 25.9 s (for a UV intensity of 500 mW/cm^2^) to 46.5 s (for a UV intensity of 100 mW/cm^2^), the falling time ranged from 112.1 s (for a UV intensity of 500 mW/cm^2^) to 168 s (for a UV intensity of 100 mW/cm^2^), which resulted in a slower recovery rather than a rising response. This asymmetric property rather limited the use of the proposed UV light intensity sensor for quasistatic applications. Although the response time exhibited a relatively slower behavior and yielded fluctuating steady-state values, it was concluded that the proposed UV light intensity sensor can overcome these technical limitations, given that the images can be acquired within a period of 3 s.

**Figure 10:**
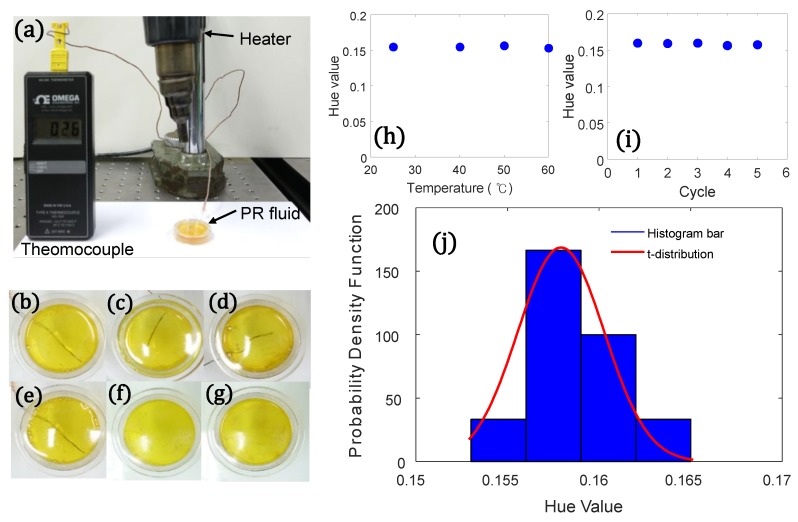
The repeatability and the effect of the temperature: (**a**) photograph of the experimental setup, (**b**) 25 °C, (**c**) 40 °C, (**d**) 50 °C, (**e**) 60 °C, (**f**) initial state at 25 °C, (**g**) recovered state after five cycles, (**h**) hue value in response to different temperatures, (**i**) hue values versus cycle, (**j**) fitted student’s *t*-distribution of hue values.

Finally, the effect of the temperature on the hue value and repeatability was examined with the use of a simple experimental setup, as shown in Figure 10a. The hue value was measured when the temperature increased from 25 °C to 60 °C, and the measurement of hue value was also repeated for five cycles at the temperature of 25 °C. The measured hue values (*N* = 10) seemed to fit a student’s *t*-distribution (*N* < 30, Freedman rule for bin size). The fitted probability density function distribution for the hue values from colorimetric responses (μ = 0.15791, σ = 5.587 × 10^−6^) demonstrated that not only the hue values were insensitive to environmental temperatures, but also the proposed UV intensity sensor showed the ability to produce the same response for the successive measurement time of 2 h. The long exposure to UV irradiation is possible with the PR fluid-based UV intensity sensor, whereas conventional UV sensors are typically not recommended for long-term continuous outdoor deployment. This attribute can be advantageous compared to the conventional UV light intensity sensors, because one of the specific applications of UV intensity sensors is the monitoring of UV radiation in outdoor environments. However, the colorimetric responses of PR fluids limit the available wavelength of UV light because PR fluids are only responsive when the wavelength of the UV light ranges from 250 nm to 380 nm. Although the proposed UV sensor can detect UV irradiation from UV-B (260–320 nm) to UV-A (320–400 nm), similar to the conventional UV sensors designed to be sensitive to UV radiation in the UV-A and UV-B ranges, the spectral range needs to be extended to the shorter wavelength range. 

## 5. Conclusions

In this study, we demonstrated that UV light-responsive capabilities can be readily imparted to PR fluids, and the colorimetric responses to different UV light intensities can be effective working principles for measuring the UV light intensity. The colorimetric response was based on the hue value of the color images, and it spanned the full range up to 2000 mW/cm^2^. The proposed UV light intensity sensor yielded a linear sensitivity response with respect to the UV light intensity. This sensor is an attractive solution for UV light intensity sensing applications because (a) only a cost-effective CMOS-type image sensor is required to detect the variation of the UV light intensity and (b) this UV intensity sensor does not require any complicated electronic circuits or MEMS technology. Further studies will focus on the miniaturization of the sensor through the implementation of a specialized embedded μ-processor suitable for only digital image processing and more compact image sensors (e.g., 4.54 × 3.42 mm for a smartphone).

## Figures and Tables

**Table 1:** List of the three primary ingredients of PR fluid samples in 100 mL of cyclohexane.

Lecithin	Sodium Deoxycholate	Spiropyran
0.1 mol	0.0398 mol	0.015 mol

## Supplementary Materials

Supplementary File 1
